# Medical Items

**Published:** 1917-11

**Authors:** 


### MEDICAL ITEMS.

Administered internally, Chloretone passes unchanged into the circulation and is deposited in considerable quantities in the cerebral tissue, the patient falling into a profound sleep. Its action is like that of natural fatigue. Hynosis passes off gradually, and no habit is formed. Acting upon the central nervous system, therapeutic doses have little or no effect upon the heart and respiratory centers.

Chloretone possesses a wide range of therapeutic applicability. It is a valuable sedative in alcoholism, cholera and colic. It is useful in epilepsy, chorea, pertussis, tetanus and other spasmodic affections. It allays, in most cases, the vomiting of pregnancy, gastric ulcer and seasickness. As a sedative and hynotic it is indicated in acute mania, puerperal mania, periodic mania, senile dementia, agitated melancholia, motor excitement of general paresis, insomnia of pain (as in tabes dorsalis, cancer and trigeminal neuralgia), insomnia of mental strain, insomnia of nervous diseases, etc. In insomnia it is often effective when other drugs have failed.

The therapeotic dose for an adult is ten to fifteen grains. Good results, however, have been had with doses as small as seven and one-half grains. Sleep usually follows in half an hour to one hour. The administration of Chloretone is not attended with digestive disturbances.

"I prescribe large quantities of Tongaline for many conditions for which it is particularly indicated, but I obtain especially gratifying results from its use for what are commonly called "colds," the symptoms of which are chills, fever, sneezing, coughing, etc., and for which the system requires, first of all, prompt and thorough elimination.

I order 4 ounces Tongaline, directing that two teaspoonfuls be taken in a hal fglass of water, preferably hot, every two hours for three or four doses or until the patient is thoroughly under the influence of the medicine; then one teaspoonful every three hours, until the attack has been absorbed or the patient has recovered.

I have personally derived great benefit from Tongaline for "colds," taking it on the first appearance of any of the symptoms, and in nearly every instance abort the disease, and I use several bottles of Tongaline myself during the winter months in that way."

"Having devoted especial attention to the treatment of chronic diseases, I think I can attribute a large part of what success I have had to the strict attention I have always given to the matter of elimination. A large percentage of such diseases have as a main factor autointoxication and the retention of morbid waste matters nature has failed to expel."

The stlicylates have long been regarded by the profession as potent anti-rheumatics, whose efficacy is unapproached by any other known medicine in the treatment of neuralgia, rheumatism, sciatica and other neurotic lesions. Tongaline, from the character of its ingredients, is bound to possess special alterative and eliminative action, with positive affinity for the excretory system of glands, necessarily producing a thorough elimination of the toxic and morbific products of the system through the various emunctories. For rheumatism and gout; sciatica and lumbago, it is the remedy par excellence; its action being absolutely invaluable.

